# Exploring performance-related inter-limb asymmetry thresholds in speed skating: a CART analysis approach

**DOI:** 10.3389/fphys.2026.1770809

**Published:** 2026-03-11

**Authors:** Zhiyong Jin, Yufeng Wang, Chunyuan Yang, Yuzi Diao, Li Yan

**Affiliations:** 1 Harbin Sport University, Harbin, China; 2 Heilongjiang Vocational College of Winter Sports, Harbin, China

**Keywords:** drop jump, limb dominance, regression tree, speed skater, squat jump

## Abstract

Long-term specialized training can lead to increased inter-limb asymmetry in speed skaters. While excessive inter-limb asymmetry may impair athletic performance, its impact on different skating phases and the corresponding thresholds remain unclear. The objective of this study was to investigate the relationship between lower limb strength asymmetry and split skating times, and to identify corresponding asymmetry thresholds using a data-driven Classification and Regression Tree (CART) analysis. Thirty-nine adolescent male speed skaters (age: 16.85 ± 0.81 years, height: 177.45 ± 4.26 cm, body mass: 66.03 ± 5.60 kg) underwent inter-limb asymmetry assessments based on single-leg vertical drop jump (SVDJ) and single-leg lateral squat jump (SLSJ) tasks. Skating performance was evaluated via a 500 m time trial, where split times for the start (20 m), straight (100 m), and curve phases (110–200 m), as well as the total time were recorded. Results showed that SVDJ jump height asymmetry explained 19.70% of the variance in 20 m start time (F(1, 37) = 9.07, p = 0.005). Moreover, in conjunction with SLSJ performance asymmetry, it explained 20.95%–24.65% of the variance in 100 m straight time (Best λ = 0.04, root mean squared error (RMSE) = 0.27) and 500 m total time (Best λ = 0.12, RMSE = 1.07). The CART analysis identified asymmetry in SVDJ jump height and SLSJ take-off velocity as the optimal variables (defined by the lowest RMSE) for distinguishing skating performance, with thresholds of 12.07% (RMSE = 0.15) and 10.26% (RMSE = 0.49), respectively. Independent samples t-tests revealed significant differences in split skating time between the high- and low-asymmetry groups defined by these thresholds (p < 0.01, Hedges’ g = 1.10–1.44). Exceeding asymmetry thresholds for SVDJ jump height and SLSJ take-off velocity may negatively affect start, straight and 500 m total skating times in adolescent male speed skaters. This inter-limb asymmetry threshold classification method may serve as a reference for performance evaluation in other sports.

## 1 Introduction

Inter-limb asymmetry is characterized by the quantification of functional or morphological discrepancies between contralateral limbs (Bishop et al., 2018; D’Hondt et al., 2025b). Previous studies have indicated that increased inter-limb asymmetry may impair sport performance such as sprinting, bilateral jumping and endurance running (Fox et al., 2023; D’Hondt et al., 2024). Although differences in assessment tasks, calculation methods, populations, and sports disciplines may have led to inconsistent results (Bishop et al., 2018), the potential risk that excessive inter-limb asymmetry may have a detrimental impact on performance has prompted practitioners to incorporate inter-limb asymmetry assessments into routine athlete monitoring protocols. However, given the intensifying nature of elite competition, training strategies must adhere to the principle of maximizing efficacy rather than solely pursuing perfect symmetry, which is rarely attainable (Maloney, 2019). Therefore, establishing specific inter-limb asymmetry thresholds associated with athletic performance is critical for facilitating more efficient and precise training prescriptions.

Conventionally, an asymmetry magnitude of 10%–15% is considered the safe threshold for athletic performance, where exceeding this threshold may result in performance impairment. However, this criterion was not derived from performance-based evidence, but was rather extrapolated from injury risk assessment protocols (Barber et al., 1990; Noyes et al., 1991; Rohman et al., 2015). Although the 10%–15% threshold is commonly used to classify athletes as ‘asymmetrical’, emerging evidence suggests that this magnitude may not significantly impair performance. For example, no significant differences were observed in change of direction (COD) and sprint performance between groups stratified by such threshold (Domínguez-Navarro et al., 2024). Virgile and Bishop (2021) highlighted that inter-limb asymmetry is task-specific, where both the magnitude and direction of asymmetry can vary across tasks and variables within the same individual, and may also be influenced by training background (Chapelle et al., 2022; D’Hondt et al., 2022). Moreover, inter-limb asymmetry assessment tasks exhibit varying degrees of sensitivity to distinct physical qualities (D’Hondt et al., 2025c). Collectively, a universal threshold is inadequate to accurately capture bilateral discrepancies across diverse neuromuscular profiles, indicating that inter-limb asymmetry thresholds should be stratified according to task and subject characteristics (Dos’Santos et al., 2021). Against this background, researchers have attempted to establish the threshold based on the samples’ mean asymmetry + 0.2 standard deviations (SD) (Lockie et al., 2014; Dos’Santos et al., 2017; DosʼSantos et al., 2018), or the mean + SD criterion (Graham-Smith et al., 2015). While these approaches have successfully incorporated subject and task specificity to formulate more targeted asymmetry thresholds, their ability to effectively differentiate athletic performance between groups appears limited (Hedges’ g ≤ 0.76) (DosʼSantos et al., 2018), indicating a need to further explore more sensitive methods for establishing performance-based inter-limb asymmetry thresholds.

Previous studies have made meaningful contributions to the identification of performance-related inter-limb asymmetry thresholds. However, whether extrapolating from injury risk experience or relying on mean and SD based calculations, these methods inherently rely on *a priori* assumptions. In this regard, data-driven approaches based on machine learning may offer a novel perspective for further exploring inter-limb asymmetry thresholds. Classification and Regression Tree (CART) analysis is a supervised machine learning algorithm, which employs non-parametric data mining techniques to recursively partition a dataset into homogeneous subsets, ultimately generating an intuitive decision tree model that elucidates complex relationships among variables (Razi and Athappilly, 2005). Specifically, this method is capable of automatically identifying specific thresholds for key predictors, determining optimal split points via node splitting to yield clear and explicit classification rules, thereby demonstrating high interpretability and practical utility (De’ath and Fabricius, 2000). While CART has been successfully applied in talent identification (Formenti et al., 2022) and the determination of inter-limb asymmetry thresholds associated with ACL injury risk (Powers and Straub, 2022), to the best of the authors’ knowledge, no study has yet investigated athletic performance-related inter-limb asymmetry thresholds using the CART algorithm.

While the establishment of task-specific inter-limb asymmetry thresholds is necessitated (Dos’Santos et al., 2021), previous studies have extensively focused on football (Mainer-Pardos et al., 2025), basketball (Bakaraki et al., 2021), and rugby (Mangine et al., 2021), which are characterized by a combination of symmetrical (sprinting) and asymmetrical (cutting) movement demands. Although chronic exposure to asymmetrical tasks may induce increased inter-limb asymmetry (Chapelle et al., 2022; D’Hondt et al., 2022), the asymmetrical actions in team sports retain a relative randomness in direction, allowing both limbs relatively balanced opportunities to engage in task execution. In contrast, while speed skating also features mixed symmetrical (straight) and asymmetrical (curve) demands, fixed counter-clockwise racing forces continuous rightward push-off during curves. This places higher loads on the left leg (De Koning et al., 1991), potentially inducing a significant left-sided dominance through long-term training (Jin et al., 2025). Additionally, speed skating can be divided into start, straight, and curve phases, each with distinct movement patterns (De Koning et al., 1995), indicating that inter-limb asymmetry effects may be phase-specific. Previous studies indicates that the single-leg lateral jump (SLSJ) shares similar movement patterns with the lateral push-off in speed skating (Zukowski et al., 2023b), while the single-leg vertical drop jump (SVDJ) reflects the lower-limb stretch-shortening cycle (SSC) capacity required for the start (De Koning et al., 1995). Therefore, the SLSJ and the SVDJ tests have been identified as key skating performance predictors, where excessive inter-limb asymmetry is considered detrimental (Jin et al., 2025). However, the specific impact of inter-limb asymmetry on different skating phases and the corresponding thresholds remain unclear. Moreover, while traditional approaches for establishing asymmetry thresholds are often predetermined or distribution-based, a data-driven machine learning approach offers the potential to provide more substantial insights for identifying these thresholds.

Therefore, the primary objectives of this study were: 1. to examine the relationship between phase-specific skating performance and the asymmetry of critical SVDJ and SLSJ variables (those significantly correlated with start, straight, curve, and total skating times) in adolescent speed skaters; and 2. to determine asymmetry thresholds using the CART analysis and compare them with traditional methods in this population. We hypothesized that asymmetry effects would vary across phases, and that CART-derived thresholds would demonstrate improved discriminative validity in distinguishing skating performance compared to traditional methods.

## 2 Materials and methods

### 2.1 Experimental approach to the problem

A cross-sectional design was used to examine the relationship between lower limb strength asymmetry and on-ice gliding performance in speed skaters, and the CART analysis was employed to identify the corresponding asymmetry thresholds. A total of three testing sessions were conducted. The first session included measurements of height and body mass, after being informed of the testing protocols and requirements, participants completed 5 familiarization trials for each jump test. The second session consisted of the SVDJ and SLSJ jump tests, and in the third session, participants underwent on-ice skating tests. Given that the SVDJ and SLSJ abilities have been demonstrated to correlate with skating performance (Jin et al., 2025), and considering the task-specific nature of inter-limb asymmetry (Bishop et al., 2021), these two tasks were selected to assess lower limb strength asymmetry. Skating performance was evaluated via a 500 m time trial on a standard 400 m track, where split times were recorded for the start (20 m), straight (100 m), and curve (110–200 m), as well as the total 500 m time. The jump tests and on-ice skating tests were separated by at least 48 h but no more than 96 h. Participants were instructed to abstain from alcohol and caffeine intake within 3 h prior to testing and to avoid high-intensity training on the day preceding each session. All tests were conducted during the same time of day (08:00–12:00) to minimize potential effects of circadian rhythms (Manfredini et al., 1998).

### 2.2 Subjects

Participants were recruited using convenience sampling from multiple speed skating teams based at the Heilongjiang Ice Training Center. Recruitment was coordinated through the respective coaching staffs. Thirty-nine professional male speed skaters voluntarily participated in this study (age: 16.85 ± 0.81 years; height: 177.45 ± 4.26 cm; body mass: 66.03 ± 5.60 kg). All participants possessed at least 4 years of prior training experience, and participated in either the National Junior Speed Skating Championships or the National U17 Speed Skating League. Participants were excluded if they had sustained musculoskeletal injury within the past 6 months or presented with injury or illness on the day of testing. This study received approval from the Institutional Ethics Committee (Ref: 2024013) and adhered to the Declaration of Helsinki. Prior to testing, written informed consent was obtained from all participants and their legal guardians following a detailed explanation of the procedures, potential benefits and risks. An *a priori* power analysis was conducted (G*Power v. 3.1.9.7) to determine the required sample size. Based on the previous study (Jin et al., 2025), which reported correlation coefficients of approximately r = 0.50 between skating performance and jump tests, a minimum of 29 participants was required to achieve a statistical power of 0.80 with a significance level alpha of 0.05. In addition, given the limited prior research on skating performance differences between high- and low-asymmetry groups, a *post hoc* power analysis was also conducted for the independent samples t-test based on the groups stratified by the CART analysis. The results indicated that this study achieved an average statistical power of 0.822.

### 2.3 Procedures

Prior to the jump tests, a standardized warm-up was performed, comprising 5 min of cycling on a cycle ergometer, 5 min of dynamic stretching (such as side lunges, lateral/forward and backward leg swings, lunge with a twist, open/close gate) and 3 min of task-specific familiarization. A 5-min rest interval was provided between the familiarization session and the formal testing. Furthermore, participants performed dynamic stretching, dry-land exercises such as jumps and low walks, and on-ice skating warm-up (5 min each) before the skating test.

#### 2.3.1 Single-leg vertical drop jump (SVDJ)

**FIGURE 1:**
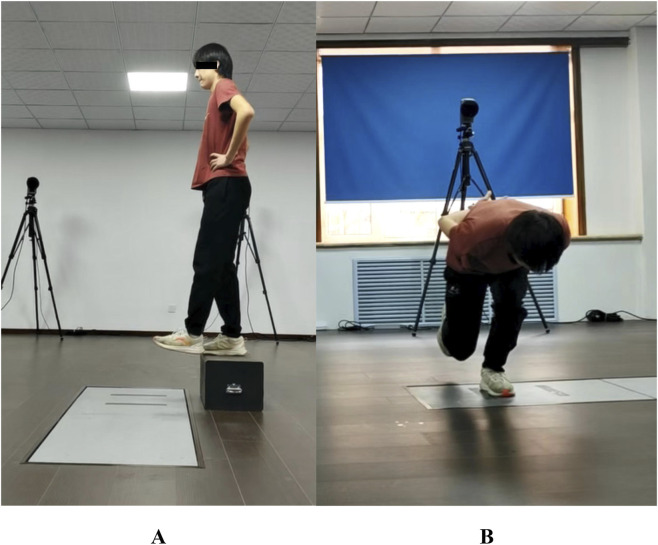
Photographic representation of the single-leg vertical drop jump **(A)** and single-leg lateral squat jump **(B)**.

Kinetic data during the take-off phase were collected using a force plate (Kistler Instrument AG, Winterthur, Switzerland) at a sampling rate of 1,000 Hz. Three cameras (SONY HDR-FX1000E, Tokyo, Japan) were positioned 5 m to the left, right, and front of the participant to record the take-off motion at 50 Hz for kinematic analysis. The optical axes of the lateral cameras were perpendicular to the participant’s sagittal plane, while the optical axis of the frontal camera was perpendicular to the frontal plane. A 24-point PEAK radial calibration frame (3 m × 3 m × 3 m) was used to calibrate the three-dimensional space of the take-off area. Upon receiving the instruction, participants stepped off a 20-cm box with the test leg to land naturally on the force plate (Figure 1A) (Holm et al., 2008), then immediately performed a vertical single-leg jump and landed on both feet. Participants were required to keep hands on their hips throughout the movement and aim to jump as high as possible while minimizing ground contact time. Trials were repeated if: 1. the participant jumped rather than stepped off the box; 2. removed hands from hips during the jump. The SVDJ task was performed on alternating legs with a randomized starting order (Lockie et al., 2014). A total of six jumps were completed (three per leg), with a 45-s rest interval between trials (Dobbs et al., 2015). The average outcomes of all 3 jumps for each leg was used for analysis.

#### 2.3.2 Single-leg lateral squat jump (SLSJ)

Following the SVDJ, the SLSJ test was conducted (Figure 1B). The acquisition of kinetic data followed the same protocol as the SVDJ. Jump distance was measured via tape measure from the black tape’s medial edge on the force plate to the nearest lateral point of the landing foot. Participants stood unilaterally on the force plate, aligning the medial border of the support foot with the inner edge of the black tape. Upon receiving the instruction, participants flexed the support knee to approximately 90° and leaned the trunk forward, with hands placed behind their back to simulate the skating posture. After holding this posture for 2 s, jumped laterally upon command and landed simultaneously on both feet. Trials were considered invalid and repeated if: 1. hands were not maintained behind the back throughout the jump; 2. the non-supporting leg touched the ground prior to take-off; 3. the feet landed sequentially; 4. the participant fell or shifted position upon landing. The rest intervals, trial order, and number of repetitions were identical to those of the SVDJ.

#### 2.3.3 Skating performance testing

The on-ice testing was conducted on a standard 400 m long-track speed skating oval equipped with an ‘intelligent ice rink’ system, which consists of 20 cameras covering the entire track and utilizes computer vision technology to capture parameters such as skating trajectory, distance, velocity, and time. Each participant completed one 500 m trial individually starting from the inner lane to avoid lane bias and drafting effects. The system recorded split times for the first 20 m (start phase), 100 m (straight phase), 110–200 m (curve phase), and the total 500 m duration for analysis. All timing data were recorded with a precision of two decimal places.

#### 2.3.4 Asymmetry calculations

The asymmetry magnitudes of the variables derived from SLSJ and SVDJ were calculated as 100 - (100/maximum value × minimum value) and expressed as a percentage, with directionality assigned using an IF function in Microsoft Excel 2016 (positive = left dominance; negative = right dominance) (Bishop et al., 2021).

### 2.4 Data processing

Kinematic parameters of the SVDJ were analyzed using SIMI Motion 8.0 (SIMI Reality Motion Systems, Germany). Seventeen anatomical points including the head and bilateral hands, elbows, shoulders, hips, knees, ankles, toes, and heels were manually digitized frame-by-frame. The Body center of gravity (BCoG) was calculated based on the Hanavan human body model (Hanavan, 1964). Raw data were smoothed using a low-pass digital filter, with residual analysis determining the optimal cut-off frequency ranging from 6 to 10 Hz (Winter, 1990). Multi-camera synchronization was achieved by aligning the event of toe-off. Three-dimensional coordinates were reconstructed using the direct linear transformation (DLT) algorithm. The manual digitization process demonstrated high reliability, with an intraclass correlation coefficient (ICC) of 0.96 for BCoG velocity (Atkinson and Nevill, 1998). Kinetic data were processed in MATLAB R2021. Raw force data were filtered using a low-pass filter with a cut-off frequency of 75 Hz. The instants of take-off and the onset of the push-off phase were defined as the points where vertical force dropped below 25 N and exceeded body weight (BW) by 10 N, respectively (Hunter et al., 2005). Consistent with previous studies for the SLSJ, peak force, rate of force development (RFD), impulse (Guo et al., 2020), and take-off velocity were calculated for both lateral (Y-axis) and vertical (Z-axis) directions based on force-time curves (Jin et al., 2025). RFD was calculated using the equation: (F_peak_ - F_initial_)/Δ T_peak_, where F_peak_ represents the peak force, F_initial_ represents the force at the onset of the push-off phase, and Δ T_peak_ represents the time duration from the onset of push-off to the instant of peak force (Jensen and Ebben, 2007). For the SVDJ task, the propulsive peak force phase and jump height were calculated based on the BCoG velocity at touchdown and force-time curves (McMahon et al., 2021). The onset of the propulsive phase was defined as the instant when BCoG velocity reached 0 m/s. Jump distance was normalized by body height (Dobbs et al., 2015), while peak force, RFD and impulse were normalized by body mass. Furthermore, in accordance with the recommendations of Holm et al. (2008), SVDJ related variables were further normalized by ground contact time to reflect SSC capabilities.

### 2.5 Statistical analysis

Analyses were conducted in SPSS 21.0 (IBM, Armonk, NY, United States). Statistical significance was set at p < 0.05. Results are presented as mean ± SD. The normality of data distribution was assessed using the Shapiro-Wilk test. Jump variables were averaged across three trials. Pearson’s correlation was used to examine the relationships of dominant limb for jump performance (the side with the better performance outcome was defined as the dominant side for each specific jump task) (D’Hondt et al., 2025a) and critical variables (jump performance variables significantly correlated with skating times) asymmetry with skating times. Paired sample t-tests were used to detect inter-limb differences, while independent sample t-tests were employed to assess differences between high and low asymmetry groups. For non-normally distributed data, Spearman’s rank correlation, the Wilcoxon signed-rank test, and the Mann-Whitney U test were utilized, respectively. Classification and regression tree (CART) analysis was utilized to construct a binary tree to determine asymmetry thresholds for critical variables, with critical variable asymmetry as the predictor and skating time as the outcome. Participants with an asymmetry magnitude above the threshold were defined as the high asymmetry group, while those below the threshold were defined as the low asymmetry group. The magnitude of correlation coefficients was interpreted according to Hopkins et al. (2009): trivial (0–0.1), small (0.1–0.3), moderate (0.31–0.5), large (0.51–0.7), very large (0.71–0.9), and almost perfect (0.91–1.0). Effect sizes (ES) for between-group differences were estimated using Hedges’ g and interpreted as: trivial (<0.20), small (0.20–0.49), medium (0.50–0.79), large (≥0.8) (Hopkins et al., 2009). Test reliability (within-session) was assessed across the three trials for each leg using the coefficient of variation (CV) and the intraclass correlation coefficient (ICC) based on a two-way random effects, absolute agreement model with 95% confidence intervals (CI). Reliability was interpreted following the guidelines of Bradshaw et al. (2010): good (ICC > 0.67 and CV < 10%), moderate (ICC < 0.67 or CV > 10%), and poor (ICC < 0.67 and CV > 10%). Furthermore, to quantify the impact of asymmetry on skating performance, asymmetry variables significantly correlated with split skating times were included in a regression analysis. Given the stringent sample size requirements of traditional multivariate statistical methods such as factor analysis (Mundfrom et al., 2005), and the inherent interdependence among the jump test variables (Turner et al., 2020), a least absolute shrinkage and selection operator (LASSO) regression was employed. This method utilizes L1 regularization to mitigate the potential effects of multicollinearity among predictors (Kolodziej et al., 2023). Additionally, although SLSJ take-off velocity and jump distance represent different manifestations of the same performance parameter, the latter was excluded from the regression model due to its height dependence (Nunes et al., 2013). Given the relatively small sample size, leave-one-out cross-validation (LOOCV) was employed to determine the optimal penalty parameter λ corresponding to the minimum mean squared error (MSE) (Przednowek et al., 2018). For the 20 m split time, ordinary least squares (OLS) regression was applied as SVDJ jump height was the sole predictor included. For the CART analysis, a regression tree model was chosen given the continuous nature of all variables. Given the small sample size, the traditional hold-out method may compromise generalization and inflate the variance of model performance evaluation. Therefore, the model was built on the full dataset and validated using LOOCV (Vabalas et al., 2019). To mitigate model overfitting caused by excessive tree growth, the maximum number of splits was pre-set to 3, and the minimum leaf size was set to 5. Optimal hyperparameters such as the maximum number of splits and minimum leaf size were further identified based on LOOCV, with model performance assessed via the root mean squared error (RMSE) (Hawkins et al., 2003). Additionally, the asymmetry thresholds identified by CART were compared with other traditional threshold determination methods such as 10%, 15%, Mean + 0.2 SD and Mean + SD. Both LASSO regression and CART analyses were performed in MATLAB R2021.

## 3 Results

The general characteristics of the SLSJ and SVDJ tests are presented in Supplementary Table S1. The reliability for jump related variables ranged from moderate to good. Significant inter-limb differences (p < 0.01) were observed in the SLSJ for jump distance, take-off velocity, lateral peak force, lateral RFD and vertical peak force with a general trend of left dominance. The asymmetry magnitude across variables ranged widely from 4.27% to 16.34% (Supplementary Table S2; Supplementary Figure S1).

In the SLSJ task, jump distance (r = −0.49, p = 0.002; r = −0.43, p = 0.006), lateral peak force (r = −0.62, p < 0.001; r = −0.63, p < 0.001), lateral RFD (r = −0.55, p < 0.001; r = −0.53, p = 0.001), lateral impulse (r = −0.63, p < 0.001; r = −0.63, p < 0.001) and takeoff velocity (r = −0.62, p < 0.001; r = −0.59, p < 0.001) demonstrated moderate to large negative correlations with 100 m and 500 m skating times, respectively (Table 1). Regarding the SVDJ task, jump height also exhibited moderate to large negative correlations with 20 m (r = −0.65, p < 0.001), 100 m (r = −0.49, p = 0.002) and 500 m (r = −0.36, p = 0.026) skating times. No significant correlations were found between the 110–200 m split time and jump performance.

**TABLE 1:** Correlation between jump tests and 500 m skating time (n = 39).

Variables	20 m	100 m	110–200 m^†^	500 m
SLSJ				
Jump distance	−0.24	−0.49**	0.10	−0.43**
Peak force-V	0.01	−0.03	0.29	0.02
RFD-V	0.06	0.06	0.23	0.12
Peak force-L	−0.28	−0.62**	0.02	−0.63**
RFD-L	−0.25	−0.55**	0.04	−0.53**
Impulse-V	−0.06	−0.13	0.24	−0.04
Impulse-L	−0.30	−0.63**	0.02	−0.63**
Take-off velocity	−0.28	−0.62**	0.10	−0.59**
SVDJ (general)				
Jump height	−0.65**	−0.49**	0.12	−0.36*
Peak force-V	−0.08	−0.06	0.23	0.04
SVDJ (time normalized)				
Jump height	−0.27	−0.06	−0.11	−0.11
Peak force-V	0.11	0.17	0.04	0.18

SLSJ, single-leg lateral squat jump; SVDJ, single-leg vertical drop jump; V, vertical; L, lateral; RFD, rate of force development; *, P < 0.05; **, P < 0.01; †, non-normally distributed variables. Pearson correlation was used for normally distributed variables, and Spearman’s rank correlation was used for those marked with †.

As shown in Table 2, 20 m skating time exhibited a moderate positive correlation with SVDJ jump height asymmetry (r = 0.44, p = 0.005). Likewise, 100 m time demonstrated moderate positive correlations with asymmetry in SVDJ jump height (r = 0.36, p = 0.023), as well as SLSJ jump distance (r = 0.34, p = 0.037), lateral impulse (r = 0.37, p = 0.011), and take-off velocity (r = 0.40, p = 0.012). Moderate correlations were also found between 500 m time and the asymmetry of SLSJ jump distance (r = 0.42, p = 0.008), lateral peak force (r = 0.37, p = 0.020), lateral impulse (r = 0.38, p = 0.016), take-off velocity (r = 0.49, p = 0.002), and SVDJ jump height (r = 0.37, p = 0.021).

**TABLE 2:** Correlation between the critical variables asymmetries and 500 m skating time (n = 39).

Distance	SVDJ (general)	SLSJ				
	Jump height^†^	Jump distance	Peak force-L	RFD-L^†^	Impulse-L	Take-off velocity
20 m	0.46**	—	—	—	—	—
100 m	0.36*	0.34*	0.27	−0.05	0.37*	0.40*
500 m	0.37*	0.42**	0.37*	−0.02	0.38*	0.49**

SLSJ, single-leg lateral squat jump; SVDJ, single-leg vertical drop jump; L, lateral; RFD, rate of force development; *, P < 0.05; **, P < 0.01; †, non-normally distributed variables. Pearson correlation was used for normally distributed variables, and Spearman’s rank correlation was used for those marked with †.

Given that SVDJ jump height was the sole critical variable for the 20 m skating time, only SVDJ height asymmetry was included as the independent variable in the regression model for this distance. Linear regression analysis revealed (Table 3) that SVDJ jump height asymmetry explained 19.70% (based on R^2^) of the variance in 20 m skating time (F(1, 37) = 9.07, p = 0.005). The LASSO regression model (Table 4) selected asymmetry in SVDJ jump height (β = 0.13), SLSJ takeoff velocity (β = 0.10), and SLSJ lateral impulse (β = 0.05) as predictors for 100 m skating time, accounting for 20.95% of the variance (Best λ = 0.04, RMSE = 0.27). Regarding the 500 m skating time, SLSJ takeoff velocity asymmetry (β = 0.45) was identified as the primary predictor, followed by SVDJ jump height asymmetry (β = 0.21) and SLSJ peak lateral force asymmetry (β = 0.12), collectively explaining 24.65% of the variance (Best λ = 0.12, RMSE = 1.07).

**TABLE 3:** Linear regression analysis of the effect of single-leg vertical drop jump height asymmetry on 20 m skating time (n = 39).

Variables	B	SE	95% CI	Std. β	t	P
Constant	3.37	0.05	[3.27, 3.47]		68.61	<0.001
SVDJ jump height asymmetry (general)	0.01	0.01<	[0.01<, 0.02]	0.44	3.01	0.005

B, unstandardized regression coefficient; SE, standard error; CI, confidence interval; Std. β, Standardized regression coefficient; SVDJ, single-leg vertical drop jump.

**TABLE 4:** Variable selected for predicting 100 m and 500 m skating time based on LASSO regression analysis (n = 39).

Variables	Model a (100 m)			Model b (500 m)		
	B	Std. β	Rank	B	Std. β	Rank
SVDJ jump height asymmetry (general)	0.02	0.13	1	0.04	0.21	2
SLSJ take-off velocity asymmetry	0.03	0.10	2	0.12	0.45	1
SLSJ impulse-L asymmetry	0.01	0.05	3	0	0	—
SLSJ peak force-L asymmetry	0	0	—	0.02	0.12	3
SLSJ RFD-L asymmetry	0	0	—	0	0	—

B, unstandardized regression coefficient; Std. β, Standardized regression coefficient. Coefficients of 0 indicate variables excluded by the model. SLSJ, single-leg lateral squat jump; SVDJ, single-leg vertical drop jump; L, lateral; RFD, rate of force development.

**FIGURE 2:**
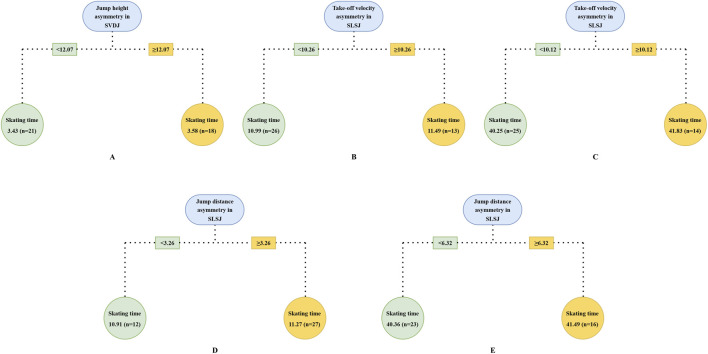
CART model with single split to discriminate skating time in different phase. The root node displays the predictor variable used for splitting, the splitting rule and corresponding threshold value are indicated along the branch lines leading to the left and right child nodes, respectively. The leaf node reports the mean skating time and sample size for subjects satisfying the corresponding branch condition. Optimal model for 20 m skating time **(A)**; optimal model for 100 m skating time **(B)**; optimal model for 500 m skating time **(C)**; CART model for 100 m skating time based on the SLSJ jump distance asymmetry **(D)**; CART model for 500 m skating time based on the SLSJ jump distance asymmetry **(E)**. SLSJ, single-leg lateral squat jump; SVDJ, single-leg vertical drop jump.

**FIGURE 3:**
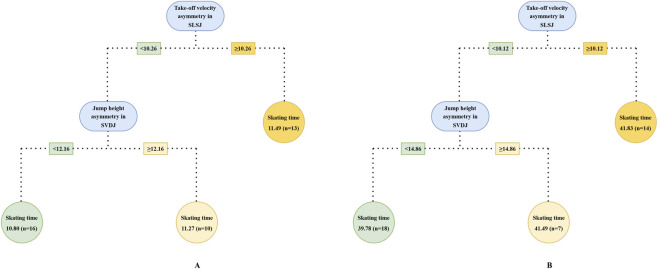
CART model with two splits to discriminate skating time in different phase. The splitting rules of the tree is the same as that of Figure 2. Optimal model for 100 m skating time **(A)**; optimal model for 500 m skating time **(B)**. SLSJ, single-leg lateral squat jump; SVDJ, single-leg vertical drop jump.

The results of the CART analysis indicated that a regression tree with a single split was sufficient to achieve a good fit across split skating times (Figure 2). SVDJ jump height asymmetry (split point: 12.07%, RMSE = 0.15) was identified as the optimal splitting variable for the 20 m model (Figure 2A), while SLSJ take-off velocity asymmetry emerged as the optimal splitting variable for both 100 m (split point: 10.26%, RMSE = 0.49) and 500 m (split point: 10.12%, RMSE = 1.19) skating times (Figures 2B,C). Additionally, SLSJ jump distance as an analogous variable to take-off velocity, also provided a reasonable fit in 100 m and 500 m regression tree models (split point: 3.26%, RMSE = 0.53; split point: 6.32%, RMSE = 1.40) (Figures 2D,E). Increasing the number of splits to 2, the models for 100 m (RMSE = 0.55) (Figure 3A) and 500 m (RMSE = 1.17) (Figure 3B) skating times revealed potential interactions among variables. Specifically, within the low SLSJ take-off velocity asymmetry subgroup, SVDJ jump height asymmetry further differentiated skating performance.

**FIGURE 4:**
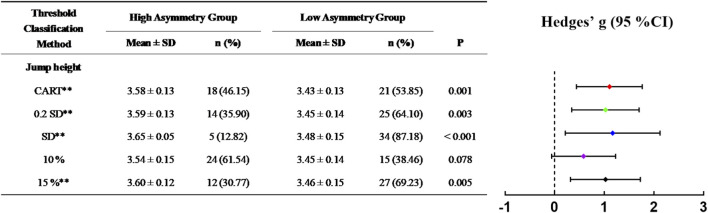
Differences in 20 m skating time between high and low asymmetry groups partitioned by different thresholds. In the forest plot, confidence intervals of Hedges’ g crossing the dashed zero line indicate no statistically significant difference between groups. CART, groups defined by the threshold determined via Classification and Regression Tree; 0.2 SD, groups defined by the threshold of mean + 0.2 standard deviations; SD, groups defined by the threshold of mean + 1 standard deviation; 10%, groups defined by a 10% threshold; 15%, groups defined by a 15% threshold; **, significant difference between groups, P < 0.01.

**FIGURE 5:**
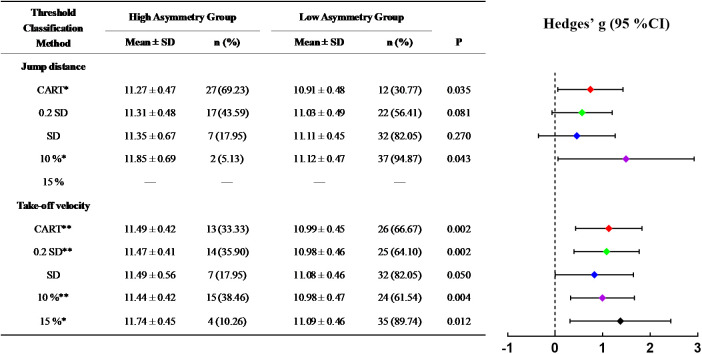
Differences in 100 m skating time between high and low asymmetry groups partitioned by different thresholds. CART, groups defined by the threshold determined via Classification and Regression Tree; 0.2 SD, groups defined by the threshold of mean + 0.2 standard deviations; SD, groups defined by the threshold of mean + 1 standard deviation; 10%, groups defined by a 10% threshold; 15%, groups defined by a 15% threshold; *, significant difference between groups, P < 0.05; **, significant difference between groups, P < 0.01.

**FIGURE 6:**
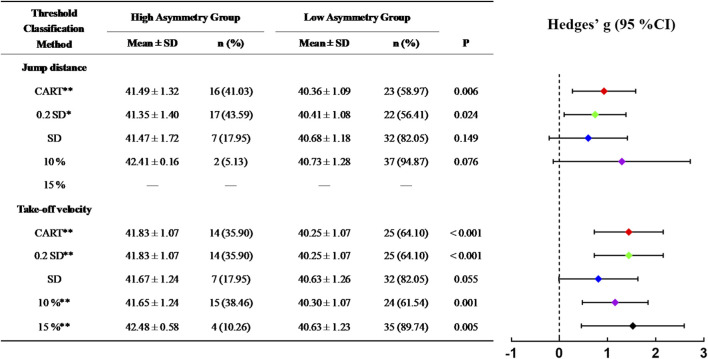
Differences in 500 m skating time between high and low asymmetry groups partitioned by different thresholds. CART, groups defined by the threshold determined via Classification and Regression Tree; 0.2 SD, groups defined by the threshold of mean + 0.2 standard deviations; SD, groups defined by the threshold of mean + 1 standard deviation; 10%, groups defined by a 10% threshold; 15%, groups defined by a 15% threshold; *, significant difference between groups, P < 0.05; **, significant difference between groups, P < 0.01.

Figures 4–6 illustrate the skating performance and sample distribution characteristics between groups stratified by different asymmetry thresholds. The CART identified thresholds distinguished groups with moderate to large differences in skating performance (ES: 0.74–1.44), partitioning the high and low asymmetry groups at the 53.85–64.10th percentiles of the sample. Compared with the CART method, 10%, 15% and Mean + SD threshold exhibited relatively smaller effect sizes or wider 95% CIs, as well as more dispersed sample percentile ranges (38.46%–94.87%). Specifically, the 15% threshold did not stratify groups for SLSJ jump distance as it exceeded the upper limit of its asymmetry range. Although the Mean + 0.2 SD method identified threshold values and sample distributions closest to the CART method, no significant difference was observed in 100 m skating time for SLSJ jump distance asymmetry (ES: 0.57, 95% CI: −0.07–1.20).

## 4 Discussion

This study aimed to investigate the effects of asymmetry in critical variables derived from SVDJ and SLSJ tasks on skating performance across different phases, and to compare differences among asymmetry threshold determination methods. The results indicated that the 110–200 m curve skating time was not associated with performance in either jump task. However, increased SVDJ jump height asymmetry exerted a negative influence on the 20 m start performance and explained 19.70% of its variance. Furthermore, together with asymmetries in SLSJ take-off velocity, lateral impulse, and lateral peak force, these variables collectively explained 20.95%–24.65% of the variance in the 100 m straight and 500 m total skating times. Furthermore, compared with other methods, the asymmetry thresholds determined by the CART method demonstrated relatively higher and more robust discriminative ability for skating performance.

Speed skating requires athletes to skate in a counter-clockwise direction around a 400 m track. The unique technique during the curve phase, which is characterized by a leftward body lean and rightward push-off by both legs, can induce asymmetrical loading on the lower limbs (De Koning et al., 1991). Consequently, long-term training may lead to sport-specific inter-limb asymmetry (Maloney, 2019). Consistent with previous study (Jin et al., 2025), participants exhibited left side dominance in SLSJ task related variables such as take-off velocity, jump distance, lateral peak force and lateral impulse (p < 0.01). However, Jin et al. (2025) did not report significant inter-limb differences in SLSJ jump distance. In their study, participants were required to land sequentially in the SLSJ task. This protocol (ICC: 0.74–0.87; CV: 2.31%–3.10%) demonstrated relatively lower test reliability than the simultaneous landing strategy (ICC: 0.87–0.91; CV: 2.26%–2.58%) employed in the present study. Contradictory results may be attributed to differences in testing protocols.

Afonso et al. (2022) suggested that asymmetry assessment should be based on tasks that replicate the specific movement patterns and neuromuscular characteristics of the sport. Therefore, critical variables significantly associated with split skating times were identified to further analyze the impact of corresponding asymmetries on skating performance. The results showed that SVDJ jump height was significantly correlated with 20 m (r = −0.65, p < 0.001), 100 m (r = −0.49, p = 0.002), and 500 m times (r = −0.36, p = 0.026), with the magnitude of correlation diminishing as the distance increased. The initial 20 m constitutes the start phase in speed skating, which shares a similar movement pattern with the sprint start and requires athletes to perform high frequency lower-limb SSC contractions (De Koning et al., 1995). Given that the DJ has been proven effective in assessing lower-limb SSC capacity, the SVDJ can reflect speed skating start capability to a certain extent, and the speed achieved in this phase consistently influences subsequent skating performance (De Koning et al., 1995). SLSJ related variables such as take-off velocity and lateral peak force, exhibited moderate to high negative correlations with 100 m and 500 m times. Consistent with previous study (Zukowski et al., 2023b), the SLSJ task effectively predicted skating performance, as its movement patterns and neuromuscular characteristics closely resemble the on-ice push-off technique. Nevertheless, no association was observed between jump performance and curve phase time. In contrast to the symmetrical push-off actions in the start and straight phases, the curve phase requires athletes to consistently perform asymmetrical rightward push-offs alternately to overcome centrifugal force (De Koning et al., 1991). Therefore, isolated jump performance may not effectively reflect the technical complexity of curve skating, and asymmetrical movement patterns in the curve phase appear less susceptible to the influence of inter-limb asymmetry (Maloney, 2019).

Based on the identified critical variables, the impact of corresponding asymmetries on split skating performance was further investigated. SVDJ jump height asymmetry was the sole predictor of start performance, accounting for 19.70% of the variance in 20 m time, aligning with the findings of Bishop et al. (2022) in soccer, where increased SVDJ jump height asymmetry impaired 10 m sprint performance. Specifically, for every 1% increase in SVDJ jump height asymmetry, the 20 m start time increased by 0.01 s. Furthermore, SVDJ jump height asymmetry was a consistent predictor for straight and total skating performance, when combined with SLSJ related asymmetries, these variables collectively explained 20.95% and 24.65% of the variance in 100 m and 500 m times, respectively. To be specific, for every 1% increase in the asymmetry of SVDJ jump height, SLSJ take-off velocity, and SLSJ lateral impulse, the 100 m skating time increased by 0.02 s, 0.03 s, and 0.01 s, respectively. As for the 500 m time, corresponding increases in SVDJ jump height, SLSJ take-off velocity, and SLSJ lateral peak force asymmetries resulted in time prolongations of 0.04 s, 0.12 s, and 0.02 s, respectively. As the total time gap between the 1st and 8th place finishers in the Beijing 2022 Winter Olympics Men’s 500 m speed skating competition was merely 0.25 s (International Skating Union, 2022), the potential performance gains from minimizing asymmetry are considered practically meaningful. Given that the velocity achieved during the start phase is highly correlated with 500 m performance (De Koning et al., 1992), SVDJ jump height asymmetry may influence overall skating performance through the start phase. Notably, although SLSJ lateral RFD was identified as a critical variable for both 100 m and 500 m skating times, its asymmetry was not significantly associated with corresponding performance. The push-off time in speed skating (1.21 s) (Iizuka and Tomita, 2024) is much longer than that of sprinting (0.11 s) (Jiménez-Reyes et al., 2022), potentially providing relatively sufficient time window for athletes to adopt coordination strategies that mitigate the effects of lower-limb RFD asymmetry (Turner et al., 2020). In contrast, asymmetries in SLSJ lateral peak force and impulse may directly influence the push-off efficiency, potentially leading to compensatory movement patterns (Yoshioka et al., 2010; 2011) or deviations in skating trajectory, thereby impairing skating performance.

In the start phase, the CART analysis identified an SVDJ jump height asymmetry threshold of 12.07% (Figure 2A), with a significant difference in 20 m time between low and high asymmetry groups (p < 0.01, g = 1.10). This threshold corresponded to the 53.85th percentile of the total sample, approximately half of the cohort was classified into the high asymmetry group. Lockie et al. (2014) identified a similar threshold (12.60%) in CMJ using the Mean + 0.2 SD method. However, no significant differences in 20 m sprint time between low and high asymmetry groups were reported in their study, despite both tasks reflecting SSC capacity. Lockie et al. did not restrict arm swing and used reach height as the outcome measure. Consequently, the disparate results may be attributed to differences in coordination strategies and upper-limb anthropometry (Guo et al., 2020). For both 100 m and 500 m skating times, the SLSJ take-off velocity asymmetry was consistently identified as the optimal splitting variable in the CART models (Figures 2B,C). Based on similar thresholds (10.26% and 10.12%), the models classified approximately one-third of the sample (at the 66.67th and 64.10th percentiles, respectively) as high asymmetry individuals. Compared to variables such as peak force and RFD, take-off velocity reflects integrative performance capabilities that share similar movement patterns and neuromuscular characteristics with the on-ice push-off movement (Jin et al., 2025). Consequently, its corresponding asymmetries may serve as the potential indicators for distinguishing 100 m and 500 m skating performance. By increasing the regression tree splitting to explore complex variable interactions, it was found that SVDJ jump height asymmetry further distinguished skating performance within the low SLSJ take-off velocity asymmetry subgroup (Figures 3A,B). Taken together with the regression results (Table 4), these findings suggest that excessive SVDJ jump height asymmetry may compromise start phase acceleration and subsequent skating performance. As noted by Dos’Santos et al. (2021), a single test or threshold may be insufficient to capture the complex athletic demands, and multi-task asymmetry assessment can provide more comprehensive information for performance evaluation. Notably, while the model fit for SLSJ jump distance asymmetry was lower than that for take-off velocity asymmetry, the identified thresholds in the 500 m model corresponded to similar sample percentiles (58.97th vs. 64.10th). This suggests that SLSJ jump distance retains a certain practical value for inter-limb asymmetry assessment when testing conditions are limited. It is noteworthy that asymmetry magnitude may fluctuate over time (Chapelle et al., 2023; D’Hondt and Chapelle, 2024). Although Zukowski et al. (2023a) reported no significant differences in the asymmetry of speed skaters throughout the season, D’Hondt and Chapelle indicated that subjects on the borderline of thresholds might be classified into different asymmetry groups across multiple testing sessions. Consequently, practitioners should consider conducting repeated inter-limb asymmetry assessments over a period of time to ensure a more objective and precise evaluation.

Comparisons with other threshold methods revealed relatively high variability in the distribution of high/low asymmetry groups based on fixed thresholds of 10% and 15%. Particularly in the case of SLSJ jump distance asymmetry, the 15% threshold failed to stratify the cohort due to exceeding the sample maximum (Figures 5, 6). Furthermore, regarding between-group time differences, fixed thresholds resulted in smaller effect sizes or relatively wide 95% CIs (Figures 4–6), which did not effectively distinguish skating performance. Similarly, 10% or 15% fixed thresholds did not distinguish athletic performance in team sports in previous studies (Kozinc and Šarabon, 2020; Domínguez-Navarro et al., 2024). D’Hondt et al. (2025c) also suggested that inter-limb asymmetry is task-specific, noting that the asymmetry magnitude varies substantially across different tests and variables, as well as being dependent on subject characteristics (Bishop et al., 2021). Therefore, fixed thresholds are difficult to adapt to diverse testing methods and populations for inter-limb asymmetry assessment. In contrast, the Mean + SD threshold is calculated based on actual sample characteristics, thereby ensuring a more stable classification rate. Although this method adopted a relatively conservative strategy to define high asymmetry individuals at the 82.05th–87.18th percentiles of the sample, its discriminant validity and stability regarding skating performance remain limited (Figures 4–6). The Mean + 0.2 SD method is considered to detect the smallest worthwhile change (Hopkins, 2004). Compared to other methods, it aligned most closely with the CART method in both threshold values and sample proportions. Nevertheless, the SLSJ jump distance threshold calculated by this method did not effectively differentiate 100 m skating performance (Figure 5). Similarly, Lockie et al. (2014) applied the Mean + 0.2 SD method to determine inter-limb asymmetry thresholds for multi-directional single-leg jump distance, but reported no significant sprint performance differences between groups. Beyond the potential influence of coordination strategies associated with unrestricted arm swing in their study (Guo et al., 2020), Bishop (2021) also noted the low sensitivity of jump distance for inter-limb asymmetry assessment. Consistent with Bishop, the asymmetry magnitude of SLSJ jump distance in the current study (5.25% ± 2.85%) was lower than that of other variables such as take-off velocity (9.39% ± 3.74%). Therefore, jump distance may not be the primary inter-limb asymmetry assessment metric when advanced equipment is available.

### 4.1 Practical applications

Practitioners can consider incorporating SVDJ and SLSJ into routine monitoring protocols for male adolescent speed skaters. The relevant metrics not only reflect specific strength qualities, but excessive asymmetries (SVDJ height asymmetry > 12.07% or SLSJ take-off velocity asymmetry > 10.12%) may impair skating performance. When testing resources are limited, jump distance may serve as a viable alternative to take-off velocity for the SLSJ assessment. For athletes exhibiting excessive asymmetry, unilateral training that replicate the direction of resistance and strength characteristics of the asymmetric task may serve as a potential strategy to mitigate such asymmetry (Bettariga et al., 2022).

### 4.2 Limitations and future directions

This study has certain limitations. The study sample was restricted to male adolescent speed skaters, and considering the specificity of inter-limb asymmetry, the current findings may be applicable primarily to homogeneous populations. Future research could further investigate inter-limb asymmetry characteristics and associated threshold differences across speed skaters of varying genders and ages. Furthermore, although the participants in the current study had generally surpassed the PHV stage (minimum age of 16 years) (Mirwald et al., 2002), the potential influence of biological maturation on the results cannot be entirely ruled out. Future studies should consider incorporating this factor to ensure rigorous control. In addition, while LOOCV and pre-pruning techniques were implemented to address the constraints of machine learning with a small sample size, future studies could expand the sample size to further enhance model stability and generalizability. Finally, future research should further investigate the impact of reducing corresponding asymmetries on skating performance based on controlled experimental designs.

## 5 Conclusion

The results demonstrate that excessive SVDJ jump height asymmetry may impair start performance, combined with SLSJ asymmetries in take-off velocity, lateral peak force, and lateral impulse, collectively explains 20.95% and 24.65% of the variation in straight and total skating performance, respectively. However, curve skating performance is not associated with jumping ability, highlighting the distinct mechanical nature of the curve phase. Finally, the CART analysis identified SVDJ jump height asymmetry and SLSJ take-off velocity asymmetry as optimal variables for distinguishing skating performance, with corresponding thresholds exhibiting effective discriminant validity. Future research may consider applying this methodology to establish sport and population specific inter-limb asymmetry thresholds, thereby providing a reference for practical training.

## Data Availability

The raw data supporting the conclusions of this article will be made available by the authors, without undue reservation.
