# Supplementary material for: Exploring performance-related inter-limb asymmetry thresholds in speed skating: a CART analysis approach
Source: Front Physiol. 2026 Mar 11;17:1770809. doi: 10.3389/fphys.2026.1770809 (PMC13013065; doi:10.3389/fphys.2026.1770809)
Supplement: Supplementary file 1 [file DataSheet1.docx]

Supplementary Material

# Supplementary Figures and Tables

## Supplementary Figures





**Supplementary Figure 1.** Radar chart depicting asymmetry in single-leg lateral squat jump and single-leg vertical drop jump. SLSJ, single-leg lateral squat jump; SVDJ, single-leg vertical drop jump; V, vertical; L, lateral; Imp, impulse; RFD, rate of force development; G, general; TN, time normalized.

## Supplementary Tables

**Supplementary Table 1.** Results of single-leg lateral squat jump and single-leg vertical drop jump test

| **Variables**  **(n = 39)** | | **SLSJ** | | |  | **SVDJ (General)** | | |  | **SVDJ (Time normalized)** | | |
| --- | --- | --- | --- | --- | --- | --- | --- | --- | --- | --- | --- | --- |
|  |  | **Mean ± SD** | **CV** | **ICC** |  | **Mean ± SD** | **CV** | **ICC** |  | **Mean ± SD** | **CV** | **ICC** |
| **Jump height (m)/ distance (constant) a** | **Left** | 0.85 ± 0.07 | 2.58 | 0.87(0.79-0.92) |  | 0.18 ± 0.02 | 4.19 | 0.88(0.81-0.93) |  | 0.44 ± 0.10 | 8.30 | 0.83(0.74-0.90) |
|  | **Right** | 0.81 ± 0.07 | 2.26 | 0.91(0.86-0.95) |  | 0.18 ± 0.03 | 4.55 | 0.90(0.84-0.94) |  | 0.43 ± 0.09 | 8.57 | 0.79(0.67-0.88) |
|  |  |  |  |  |  |  |  |  |  |  |  |  |
| **Peak force-V (N/kg) a** | **Left** | 13.49 ± 0.66 | 2.42 | 0.73(0.59-0.84) |  | 24.67 ± 3.44 | 4.50 | 0.87(0.79-0.92) |  | 65.41 ± 14.82 | 9.78 | 0.78(0.66-0.87) |
|  | **Right** | 13.01 ± 0.80 | 2.38 | 0.82(0.72-0.89) |  | 24.77 ± 3.73 | 5.22 | 0.85(0.77-0.91) |  | 63.95 ± 13.81 | 8.97 | 0.80(0.69-0.88) |
|  |  |  |  |  |  |  |  |  |  |  |  |  |
| **RFD-V**  **((N/s)/kg)** | **Left** | 4.27 ± 1.09 | 13.49 | 0.70(0.60-0.83) |  | — | — | — |  | — | — | — |
|  | **Right** | 4.32 ± 1.08 | 11.91 | 0.74(0.61-0.84) |  | — | — | — |  | — | — | — |
|  |  |  |  |  |  |  |  |  |  | — | — | — |
| **Peak force-L**  **(N/kg) a** | **Left** | 7.22 ± 0.82 | 3.20 | 0.90(0.83-0.94) |  | — | — | — |  | — | — | — |
|  | **Right** | 6.52 ± 0.94 | 3.43 | 0.93(0.87-0.96) |  | — | — | — |  | — | — | — |
|  |  |  |  |  |  | — | — | — |  | — | — | — |
| **RFD-L**  **((N/s)/kg) a** | **Left** | 7.86 ± 1.34 | 7.32 | 0.78(0.67-0.87) |  | — | — | — |  | — | — | — |
|  | **Right** | 8.68 ± 1.50 | 8.89 | 0.71(0.57-0.82) |  | — | — | — |  | — | — | — |
|  |  |  |  |  |  | — | — | — |  | — | — | — |
| **Impulse-V**  **(Ns/kg)** | **Left** | 0.83 ± 0.15 | 9.02 | 0.71(0.57-0.82) |  | — | — | — |  | — | — | — |
|  | **Right** | 0.84 ± 0.22 | 12.04 | 0.76(0.63-0.85) |  | — | — | — |  | — | — | — |
|  |  |  |  |  |  | — | — | — |  | — | — | — |
| **Impulse-L**  **(Ns/kg) a** | **Left** | 2.57 ± 0.18 | 2.95 | 0.79(0.67-0.88) |  | — | — | — |  | — | — | — |
|  | **Right** | 2.29 ± 0.23 | 3.70 | 0.83(0.74-0.90) |  | — | — | — |  | — | — | — |
|  |  |  |  |  |  | — | — | — |  | — | — | — |
| **Take-off velocity (m/s) a** | **Left** | 2.70 ± 0.17 | 1.95 | 0.89(0.81-0.93) |  | — | — | — |  | — | — | — |
|  | **Right** | 2.45 ± 0.22 | 2.76 | 0.88(0.81-0.93) |  | — | — | — |  | — | — | — |

SLSJ, single-leg lateral squat jump; SVDJ, single-leg vertical drop jump; V, vertical; L, lateral; RFD, rate of force development; CV, coefficient of variation; ICC, intra-class correlation coefficient; a, significant bilateral differences in SLSJ (P < 0.01).

**Supplementary Table 2.** Asymmetry magnitude of SLSJ and SVDJ tests

| **Variables**  **(n = 39)** | **SLSJ** |  | **SVDJ**  **(General)** |  | **SVDJ**  **(Time-normalized)** |
| --- | --- | --- | --- | --- | --- |
| **Jump height/ distance** | 5.25 ± 2.85 |  | 11.88 ± 6.04 |  | 14.62 ± 10.79 |
| **Peak force-V** | 4.27 ± 3.49 |  | 7.27 ± 4.55 |  | 14.02 ± 8.73 |
| **RFD-V** | 16.82 ± 11.28 |  |  |  |  |
| **Peak force-L** | 9.95 ± 5.57 |  |  |  |  |
| **RFD-L** | 14.55 ± 7.44 |  |  |  |  |
| **Imp-V** | 16.34 ± 12.79 |  |  |  |  |
| **Imp-L** | 10.97 ± 4.28 |  |  |  |  |
| **Take-off velocity** | 9.39 ± 3.74 |  |  |  |  |

SLSJ, single-leg lateral squat jump; SVDJ, single-leg vertical drop jump; V, vertical; L, lateral; Imp, impulse; RFD, rate of force development.
